# Effect of Intrathecal Baclofen on Pain and Quality of Life in Poststroke Spasticity: A Randomized Trial (SISTERS)

**DOI:** 10.1161/STROKEAHA.118.022255

**Published:** 2018-08-14

**Authors:** Michael Creamer, Geoffrey Cloud, Peter Kossmehl, Michael Yochelson, Gerard E. Francisco, Anthony B. Ward, Jörg Wissel, Mauro Zampolini, Abdallah Abouihia, Alessandra Calabrese, Leopold Saltuari

**Affiliations:** 1 From the Central Florida Pain Relief Centers, Orlando (M.C.); 2 St. George’s University Hospitals NHS Foundation Trust, London, United Kingdom (G.C.); 3 Department of Neurology, Alfred Health, Melbourne, VIC, Australia (G.C.); 4 Kliniken Beelitz GmbH Neurologische Rehabilitationsklinik, Beelitz-Heilstätten, Germany (P.K.); 5 MedStar National Rehabilitation Hospital, Washington, DC (M.Y.); 6 Shepherd Center, Atlanta, GA (M.Y.); 7 Department of Physical Medicine and Rehabilitation, University of Texas Health Science Center and TIRR Memorial Hermann Hospital, Houston (G.E.F.); 8 North Staffordshire Rehabilitation Centre, Haywood Hospital, Stoke-On-Trent, United Kingdom (A.B.W.); 9 Neurological Rehabilitation and Physical Therapy, Department of Neurology, Vivantes Hospital Spandau, Berlin, Germany (J.W.); 10 USL Umbria 2, Department of Rehabilitation, Ospedale di Foligno, Perugia, Italy (M.Z.); 11 Neuromodulation Clinical, Medtronic International, Tolochenaz, Switzerland (A.A., A.C.); 12 Abteilung für Neurologie Landeskrankenhaus Hochzirl, Austria (L.S.); 13 Research Unit for Neurorehabilitation South Tyrol, Bolzano, Italy (L.S.).; (UZ Gent, Gent, Belgium); (UZ Gent, Gent, Belgium); (Design Neuroscience Center, Doral, FL); (University of Miami, Miami, FL); (Hospital Universitario La Paz, Madrid, Spain); (Therapiezentrum Burgau, Burgau, Germany); (AZ Maastricht, Maastricht, the Netherlands and Hôpital fribourgeois Meyriez-Murten, Switzerland); (Saint Alphonsus Regional Medical Center, Boise, ID and Texas Children’s Hospital - The Woodlands, TX); (UCL Saint-Luc, Brussels, Belgium); (Rhein-Sieg-Klinik Dr Becker Klinikgesellschaft, Nümbrecht, Germany); (Villa Beretta, Costamasnaga, Lecco, Italy); (Tallahassee Neurological Clinic, Tallahassee, FL); (Univerzitetni Rehabilitacijski Institut Soca, Ljubljana, Slovenia); (St George’s Hospital, Tooting, London, United Kingdom); (Landeskrankenhaus Hochzirl, Zirl, Austria)

**Keywords:** baclofen, muscle spasticity, pain, quality of life, stroke

## Abstract

**Background and Purpose—:**

Intrathecal baclofen (ITB) is an effective treatment for managing patients with severe poststroke spasticity, who can experience continued pain and decline in their quality of life (QoL). SISTERS (Spasticity In Stroke–Randomized Study) was a randomized, controlled, open-label, multicenter, phase 4 study to evaluate ITB therapy versus conventional medical management (CMM) with oral antispastic medications for treatment of poststroke spasticity.

**Methods—:**

Poststroke patients with spasticity in ≥2 extremities and an Ashworth Scale score of ≥3 in ≥2 affected lower extremity muscle groups were randomized (1:1) to ITB (N=31) or CMM (N=29). Both treatment arms received physiotherapy throughout. The primary outcome was the change in average Ashworth Scale score in the lower extremities of the affected side from baseline to month 6. Here, we report results for secondary outcomes: pain via the Numeric Pain Rating Scale, health-related QoL by the EuroQol–5 dimensional 3 level utility score and health status visual analog scale score, stroke-specific QoL, and patient satisfaction. Analyses were performed on an intention-to-treat basis.

**Results—:**

We observed significant treatment effects in favor of ITB over CMM for changes from baseline to month 6 in Numeric Pain Rating Scale scores for actual pain (ITB versus CMM: mean, −1.17 [SD, 3.17] versus 0.00 [3.29]; median, −1.00 versus 0.00; *P*=0.0380) and least pain (mean, −1.61 [2.29] versus 0.24 [3.07]; median, −1.00 versus 0.00; *P*=0.0136), and EuroQol–5 dimensional 3 level utility scores (mean, +0.09 [0.26] versus +0.01 [0.16]; median, +0.07 versus 0.00; *P*=0.0197). Between-group differences were not statistically significant for EuroQol–5 dimensional 3 level visual analog scale, stroke-specific QoL summary, or Numeric Pain Rating Scale worst pain scores, although ITB patients showed greater numeric improvements from baseline during follow-up. More ITB patients than CMM patients (73% versus 48%) were satisfied with the spasticity reduction at month 6.

**Conclusions—:**

These data support that ITB therapy is associated with improvements in pain and QoL in poststroke patients.

**Clinical Trial Registration—:**

URL: https://www.clinicaltrials.gov. Unique identifier: NCT01032239.

## Abstract 2

Supplemental Digital Content is available in the text.

Stroke is a major cause of pain and disability among adults, resulting in a wide range of physical, emotional, and socioeconomic consequences. The cause of poststroke pain is diverse and may be related to musculoskeletal, central neuropathic causes, or because of the discomfort from increased muscle tone from spasticity.^1^ Patient quality of life (QoL) is often negatively impacted by the sequelae of a stroke; the cause is also multifaceted and seems to be directly linked to reduced mobility, inability to perform self-care and activities of daily living, and reduced social interaction,^2^ as well as subjective symptoms such as pain, fatigue, and depression.^3^

Spasticity is a relatively common long-term complication of stroke, with up to 13% of poststroke patients experience severe disabling spasticity.^4,5^ Patients with poststroke spasticity (PSS) tend to experience a higher incidence of pain and lower health-related QoL compared with patients with normal muscle tone.^6,7^ Along with physical and rehabilitation strategies, several pharmacological treatments are used for management of PSS, most commonly oral antispastic agents (eg, baclofen). Unlike systemic antispastic agents, injectable botulinum toxins are mostly used for treatment of focal spasticity.^8,9^ Intrathecal baclofen (ITB) therapy is indicated for use in severe, chronic spasticity of cerebral or spinal origin, including generalized spastic hypertonia after stroke. In the United States alone, the PSS population estimated to benefit from ITB therapy is 442 000.^5^ Limited evidence from prior nonrandomized studies and case series of stroke patients suggests that as well as reducing muscle tone and spastic hypertonia in the extremities, ITB may also reduce pain^10–12^ and improve QoL measures.^11,13^ In addition, it seems to be effective in reducing pain and improving QoL in other causes, including cerebral palsy^14,15^ and multiple sclerosis.^16–18^

To further evaluate the utility of ITB therapy for treatment of PSS, we conducted SISTERS (Spasticity In Stroke–Randomized Study), the first randomized controlled trial of ITB therapy in PSS to date. The study included 60 patients with severe PSS who had not reached their therapy goal with other treatment interventions (eg, physiotherapy, botulinum toxin injection, and oral medication) and compared outcomes with ITB therapy to those with conventional medical management (CMM) with oral antispastic medications. In line with current standards of care, both treatment arms received physiotherapy throughout the study. Results for the primary outcome, published previously,^19^ showed a significant effect of treatment with ITB therapy over CMM for reduction of spasticity in the lower limb measured with the Ashworth Scale over 6-month follow-up. Furthermore, although the study was not powered for analysis of secondary outcomes, we observed a statistically significant treatment effect for ITB over CMM for spasticity reduction in the upper limb, indicating a substantial and broad effect of ITB therapy in PSS control. In this article, we present the results for other prespecified secondary outcomes of SISTERS, including assessment of pain and QoL measures, and patient satisfaction with therapy.

## Methods

Requests to access the data set from qualified researchers trained in human subject confidentiality protocols may be sent to Medtronic at abdallah.abouihia@medtronic.com. All analytic methods have been provided with the published article.

### Study Design and Participants

SISTERS was a phase 4, randomized, controlled, open-label, parallel-group, multicenter study conducted in rehabilitation hospitals at 11 centers across Europe (Austria, Belgium, Germany, Italy, the Netherlands, Spain, United Kingdom, and Slovenia) and 7 centers in the United States between December 16, 2009, and September 21, 2016. The study was approved by the institutional review board/ethics committee and competent authorities (Europe only) at each participating site/country. Patients or their legal guardian provided written informed consent before study participation.

Full details on the design of SISTERS, including the sample size calculation and randomization methods, have been reported previously.^19^ The study evaluated the efficacy and safety of ITB therapy compared with CMM with oral antispastic medications on severe spasticity in poststroke patients after 6 months active treatment. For the purposes of this study, a stroke was defined as any acute vascular accident (ischemic or hemorrhagic). Key eligibility criteria included age 18 to 75 years, poststroke duration >6 months, and spasticity in at least 2 extremities and an Ashworth Scale score of ≥3 in at least 2 affected muscle groups in the lower extremities. Patients were excluded if they had known baclofen hypersensitivity, uncontrolled refractory epilepsy, systemic infection, cardiac pacemaker or other implantable device, or use of botulinum toxin within the 4 months before study start. The full list of study inclusion/exclusion criteria is provided in the online-only Data Supplement (Table I in the online-only Data Supplement).

Eligible patients were randomized 1:1 to receive ITB therapy or CMM via an interactive web-based system and according to a computer-generated sequence, stratified by clusters of centers via the block permutation method (randomly selected block sizes of 2 or 4).

### Treatment and Procedures

The study comprised a run-in phase (21 days for CMM arm and 2–25 days for ITB arm), followed by a 6-month active trial. Both treatment arms received physiotherapy throughout the study according to a protocol that was predefined at each center. Scheduled follow-up visits in the active phase were at week 6 (ITB arm only), month 3, and month 6.

Lioresal Intrathecal (baclofen injection; Novartis [Europe]/Saol Therapeutics [United States]) was used for ITB therapy. Patients in the ITB arm underwent an ITB test between days 1 to 10 to evaluate drug response. Patients meeting the ITB test success criterion (1-point drop in Ashworth Scale score in 3 muscle groups in the affected lower extremity) were implanted with the marketed SynchroMed II infusion system (Medtronic Inc) within 15 days of the test; patients could continue their oral antispastic medications during the run-in phase. ITB dose titration was done over the first 6 weeks of the active trial to determine the optimal therapeutic ITB dose, and oral antispastic medications were gradually tapered and discontinued by the titration period end. Patients randomized to ITB who were not implanted were followed per protocol, remaining on oral antispastic medications and physiotherapy.

Patients in the CMM arm received a combination of oral antispastic medication (at least one of oral baclofen, tizanidine, diazepam or other benzodiazepines, or dantrolene) and physiotherapy. Oral antispastic medications and doses were adjusted as deemed necessary by the investigator and in accordance with usual clinical practice and the needs of each patient.

### Outcome Assessments

Results for the study’s primary outcome (spastic hypertonia and muscle tone in the affected lower limb, as assessed by the change in average Ashworth Scale score from baseline to month 6) and safety have been reported previously.^19^

Secondary outcome measures reported here (all patient self-assessed) include pain intensity for actual, least, and worst pain (spasticity- or spasm-related for each) by the Numeric Pain Rating Scale (NPRS; range 0=no pain to 10=worst possible pain^20^), health-related QoL via the EuroQol–5 dimensional 3 level (EQ-5D-3L^21^) and stroke-specific QoL (SS-QOL^22^) instruments, and patient therapy satisfaction. Absolute and relative changes in pain were assessed. The EQ-5D-3L output included a descriptive profile (3 response categories for mobility, self-care, ability to undertake usual activities, pain, and anxiety/depression), a weighted health index utility score (computed using UK utility values^23^), and overall health status derived from a visual analog scale (EQ-5D-VAS; range 0–100, where 100=best possible health status). The SS-QOL summary score was computed as an unweighted average of the 12 domain scores, with a higher score indicating better QoL. Satisfaction with therapy was evaluated via patients’ responses to 2 statements provided in their native language (I am satisfied with the reduction in spasticity provided by my treatment; and I would recommend this therapy to a friend), each assessed with a 5-point Likert scale (1=strongly disagree; 5=strongly agree). NPRS scores and QoL measures were assessed at baseline and months 3 and 6, and patient satisfaction at months 3 and 6.

### Statistical Analysis

All reported efficacy analyses were based on the intent-to-treat population which consisted of all patients as randomized. Absolute changes in pain were assessed using NPRS_6 months_−NPRS_baseline_. Relative percentage changes were assessed using [(NPRS_6 months_−NPRS_baseline_)/NPRS_baseline_]*100. Because of nonnormal distribution of data for NPRS and EQ-5D-3L utility scores, changes from baseline to month 6 were compared between ITB and CMM arms using a nonparametric test (Wilcoxon rank-sum). Changes in EQ-5D-3L VAS and SS-QOL summary scores were compared using a pooled Student *t* test (this assumed similar sample variance for both treatments). Last observation carried forward imputation was used for analysis of all quantitative variables; patients with data missing for both months 3 and 6 were excluded from the analysis. For analysis of patient satisfaction, responses were grouped into the following 3 categories: disagree (Likert scale score of 1 or 2); neutral (3); and agree (4 or 5). All statistical tests were performed at the 2-sided α level of 5%, with no adjustment of type 1 error because of multiplicity. SAS version 9.4 was used for all statistical analyses.

## Results

### Study Population

**Figure 1.**
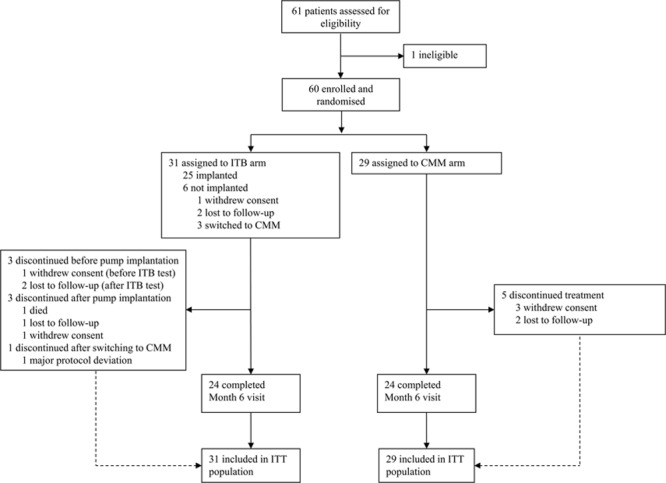
Patient flow diagram. CMM indicates conventional medical management; ITB, intrathecal baclofen; and ITT, intent-to-treat. Reproduced from Creamer et al^19^ with permission. Copyright ©2018, BMJ Publishing Group Ltd.

In total, 60 patients were enrolled and randomized (31 to ITB therapy and 29 to CMM) and were included in the intent-to-treat analysis population (Figure 1). Twenty-five patients in the ITB arm were implanted with the device; of the 6 who were not implanted, 1 withdrew consent before the ITB test, 2 were lost to follow-up after the ITB test, and 3 were switched to CMM as previously described.^19^ Twelve patients (7/31 [23%] and 5/29 [17%] in the ITB and CMM arms, respectively) discontinued prematurely and 48 patients completed follow-up at month 6 (24/31 [77%] in the ITB arm, including 22 implanted patients, versus 24/29 [83%] in the CMM arm). One ITB-implanted patient died after week 6 because of an unrelated cause (neither study drug nor study device). As shown in the Table, baseline demographic and disease characteristics were broadly similar for the 2 treatment arms. Compared with the CMM arm, both mean and median scores for NPRS actual pain were higher in the ITB arm.

**Table 1.**
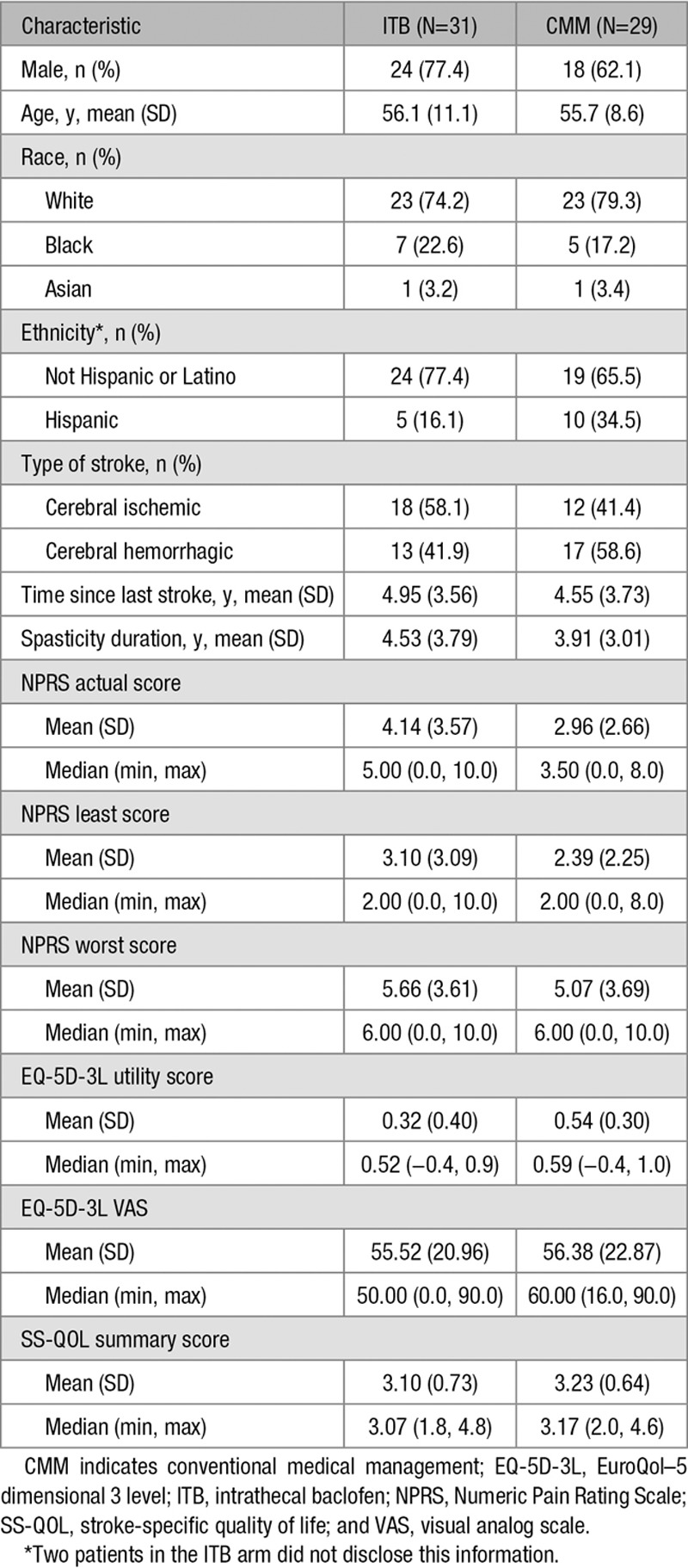
Baseline Characteristics of the Intent-to-Treat Population

### Pain Assessed by NPRS

**Figure 2.**
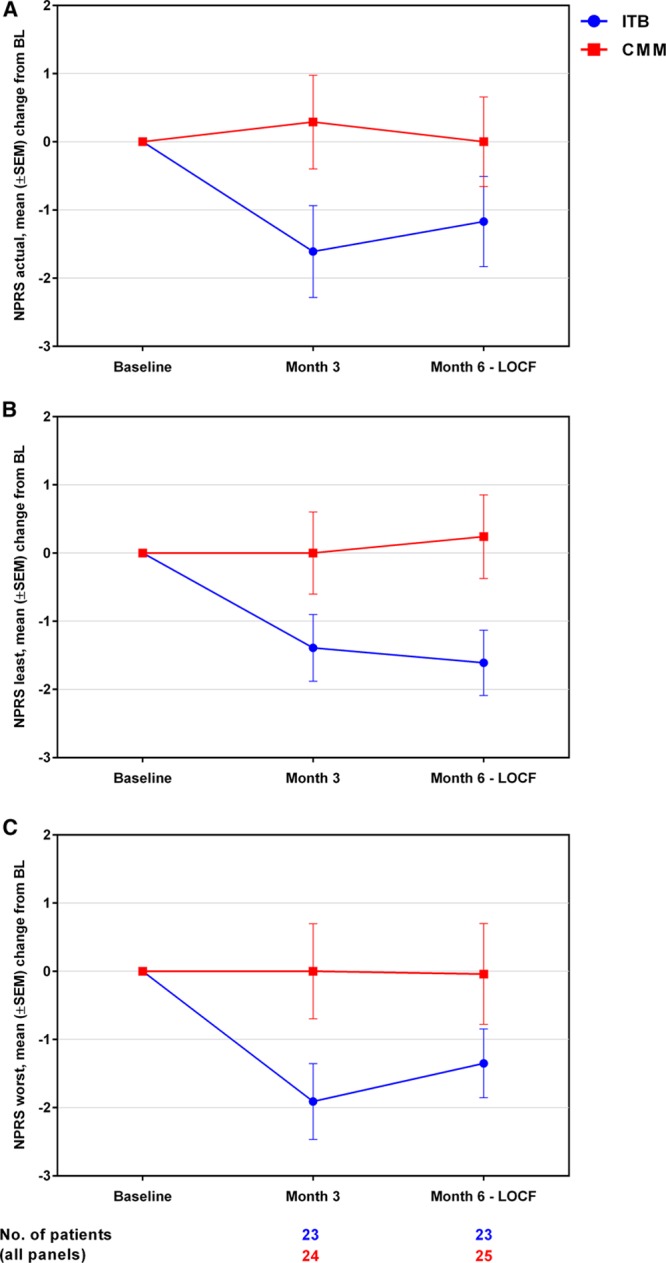
Mean (SEM) changes from baseline (BL) in Numeric Pain Rating Scale (NPRS) pain scores by visit for (**A**) actual pain, (**B**) least pain, and (**C**) worst pain related to spasticity or spasm. Last observation carried forward (LOCF) imputation was performed for the month 6 assessment using month 3 data. CMM indicates conventional medical management; and ITB, intrathecal baclofen.

Mean scores for all 3 pain ratings (actual, least, and worst) tended to decrease (improve) for the ITB arm during the follow-up period, particularly between baseline and month 3, whereas values for the CMM arm remained relatively constant (Figure 2). The analysis showed a significant treatment effect in favor of ITB therapy over CMM for the change from baseline to month 6 in actual pain (ITB versus CMM: mean change, −1.17 [SD, 3.17] versus 0.00 [3.29], and median change, −1.00 versus 0.00; *P*=0.0380) and in least pain (mean change, −1.61 [2.29] versus +0.24 [3.07], and median change, −1.00 versus 0.00; *P*=0.0136). For worst pain, a decrease in the mean score from baseline to month 3 was observed in the ITB arm, although the apparent improvement from baseline was less pronounced at month 6 (ITB versus CMM: mean change, −1.35 [2.42] versus −0.04 [3.69], and median change, 0 for both arms). The between-group difference for change in worst pain at month 6 was not statistically significant (*P*=0.2427). In the ITB arm, mean (SD) relative percentage decreases at 6 months in actual, least, and worst pain were 26.84% (32.06), 29.61% (42.29), and 18.16% (33.61) compared with relative increases of 9.63% (96.95), 12.97% (90.50), and 1.94% (87.80) in the CMM arm.

We also analyzed oral pain medication use in patients considered in the statistical analysis of NPRS scores (ie, those with NPRS data for baseline and month 3 or 6 visits). A total of 8 (of 23) ITB patients were receiving pain medication at baseline compared with 5 (of 25) CMM patients. At the month 6 visit, 5 and 4 patients increased their medication relative to baseline in the ITB and CMM arms, respectively. Two patients in each treatment arm decreased their medication before the last follow-up. The remaining patients did not show any change between baseline and month-6 assessments.

### QoL Assessed by EQ-5D-3L and SS-QOL

**Figure 3.**
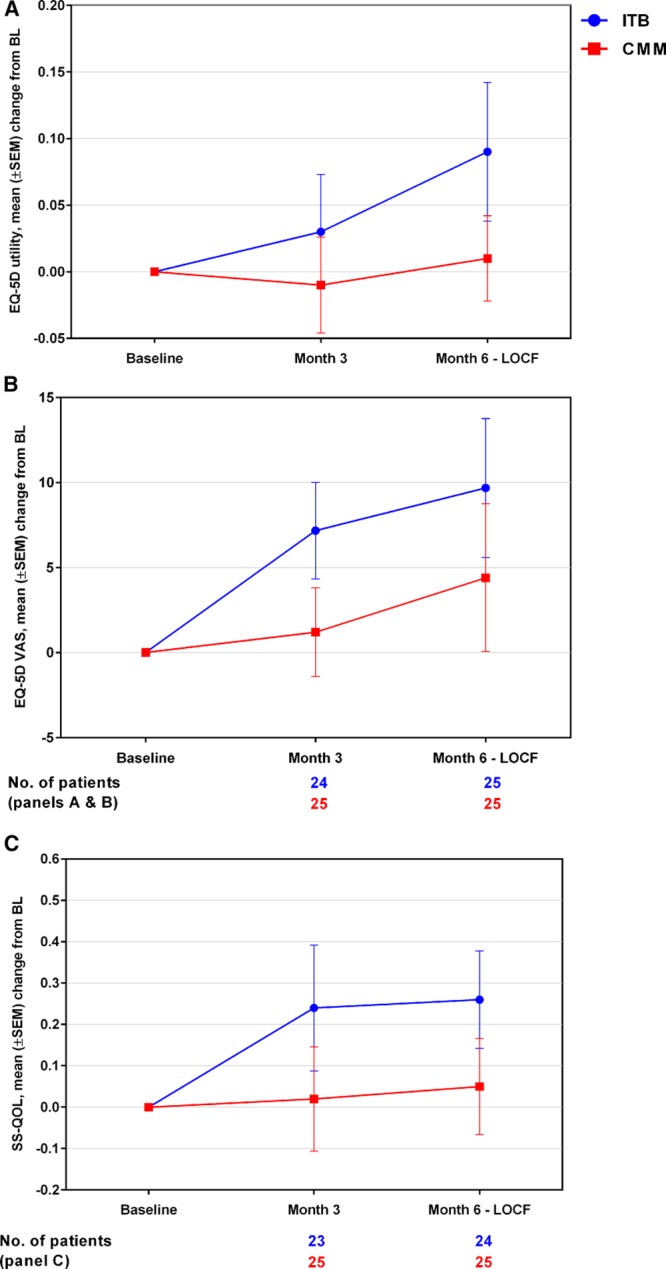
Mean (SEM) changes from baseline (BL) by visit for (**A**) EuroQol–5 dimensional 3 level (EQ-5D-3L) utility index, (**B**) EQ-5D-3L visual analog scale (VAS), and (**C**) stroke-specific quality of life (SS-QOL) summary scores. Last observation carried forward (LOCF) imputation was performed for the month 6 assessment using month 3 data. CMM indicates conventional medical management; and ITB, intrathecal baclofen.

**Figure 4.**
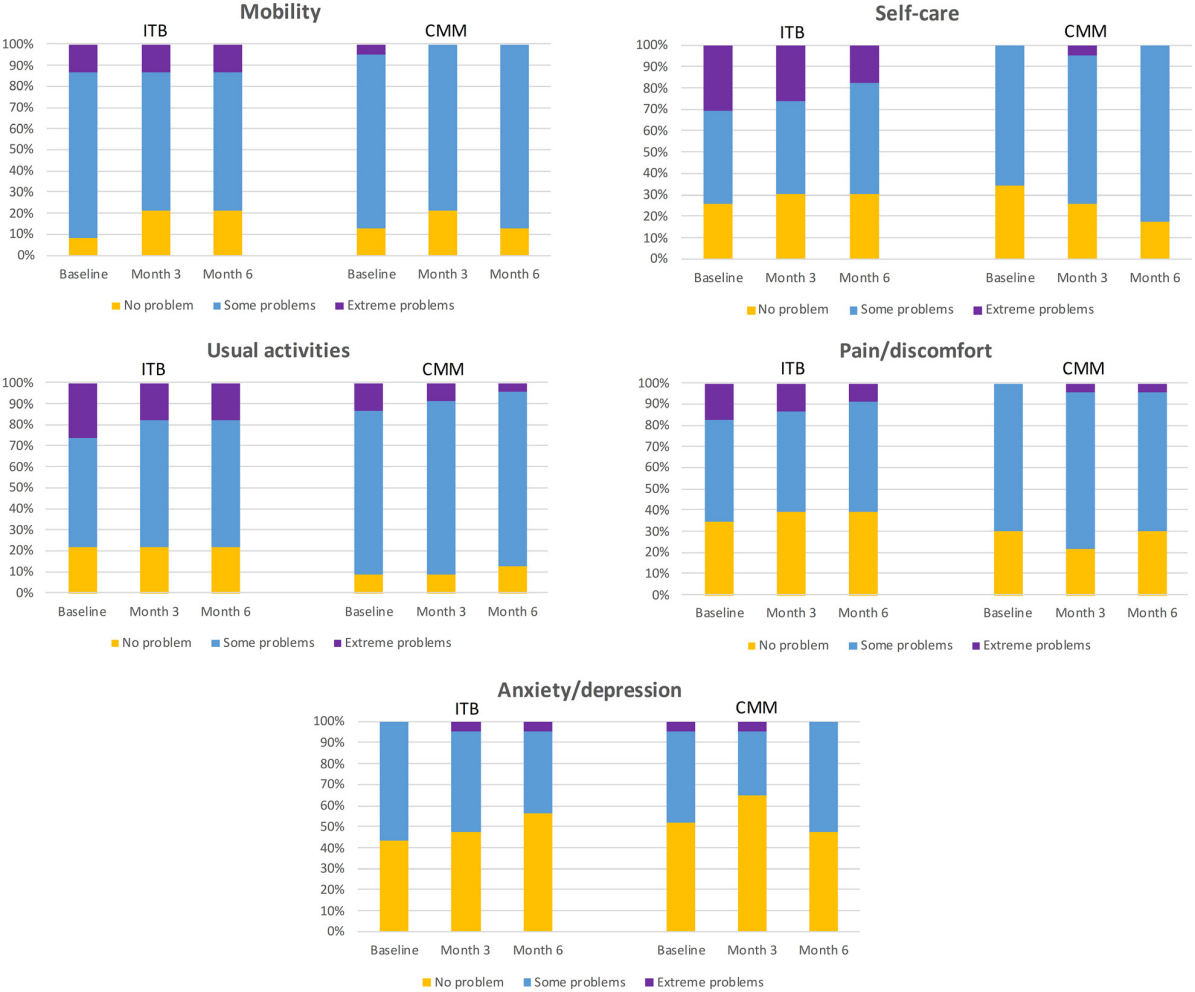
Distribution of EuroQol–5 dimensional 3 level (EQ-5D-3L) responses (percent of patients on each level of problem) for individual dimensions by visit. Only patients with data for baseline, month 3, and month 6 visits were included (N=23 for both treatment arms). CMM indicates conventional medical management; and ITB, intrathecal baclofen.

About the EQ-5D-3L utility score (Figure 3A), an increase (improvement) from baseline to month 6 was observed in the ITB arm, whereas little change was observed in the CMM arm (ITB versus CMM: mean change, +0.09 [SD, 0.26] versus +0.01 [0.16], and median change, +0.07 versus 0.00), and a significant treatment effect in favor of ITB therapy over CMM was observed (*P*=0.0197). For the EQ-5D-3L VAS (Figure 3B), an increase (improvement) from baseline to month 6 was observed in both treatment arms, with a slightly greater increase observed in ITB patients (mean change, +9.68 [20.42] for ITB and +4.40 [21.75] for CMM); the analysis showed no statistically significant difference between the 2 treatment arms (*P*=0.3807). As shown in Figure 4, imbalances between the 2 treatment arms were observed with respect to the baseline distribution of responses for individual EQ-5D dimensions, with a higher proportion of ITB arm patients reporting extreme problems for self-care (7/23 [30%] versus 0/23 [0%] for ITB and CMM, respectively), usual activities (6/23 [26%] versus 3/23 [13%]), pain/discomfort (4/23 [17%] versus 0/23 [0%]), and mobility (3/23 [13%] versus 1/23 [4%]). In the ITB arm, there was an overall decrease from baseline to month 6 in the percentage of patients reporting extreme problems for all domains except mobility and anxiety/depression. In the CMM arm, apart from a decrease in patients reporting extreme problems in usual activities, no other discernible changes in response distributions were observed during follow-up. For the SS-QOL summary score, an increase (ie, improvement) from baseline to month 6 was observed in the ITB arm, whereas little change was observed in the CMM arm (mean changes, +0.26 [0.58] for ITB and +0.05 [0.58] for CMM; Figure 3C). The difference between groups was not statistically significant for SS-QOL (*P*=0.2105).

### Patient Satisfaction

**Figure 5.**
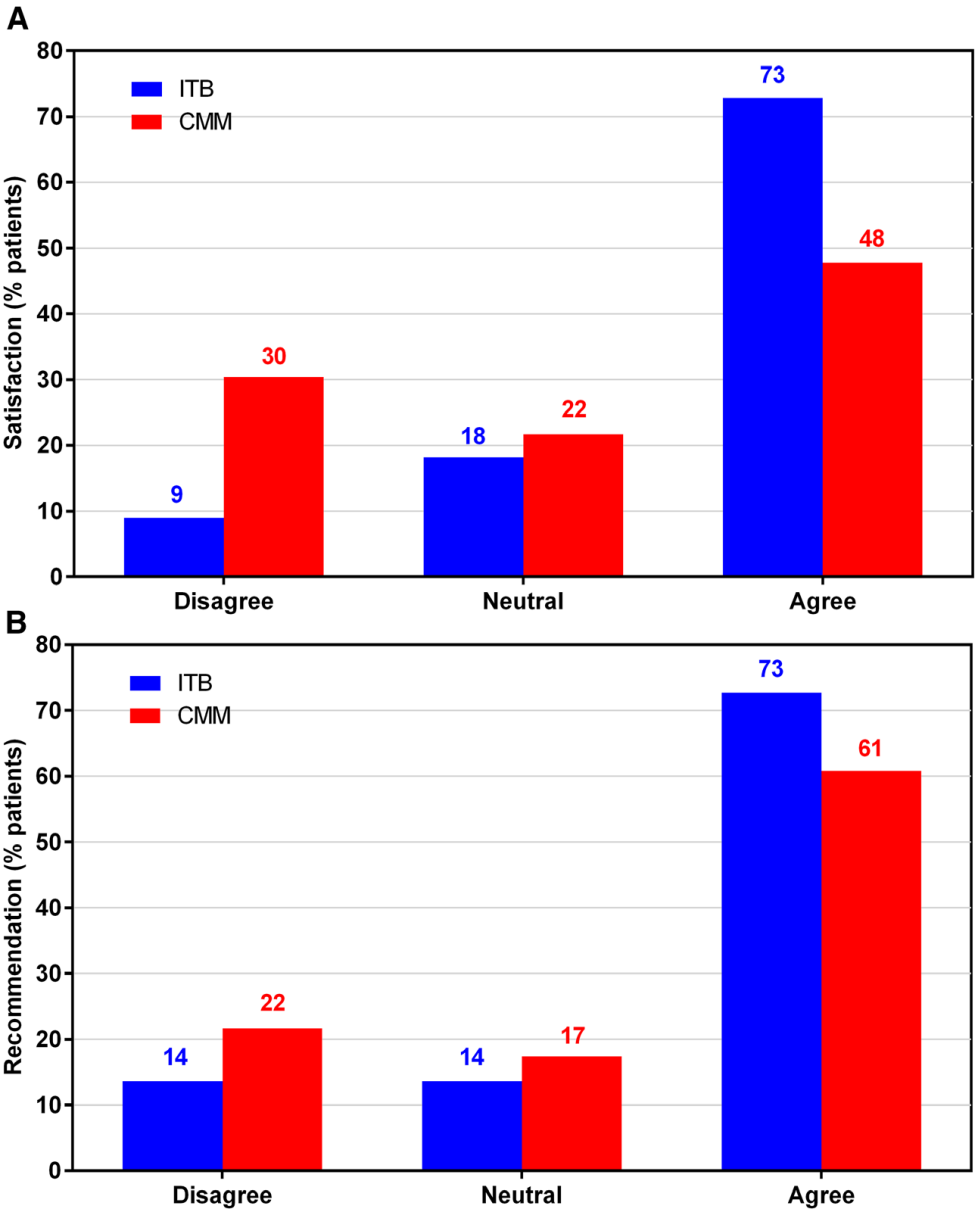
Patient satisfaction with the reduction of spasticity provided by the therapy (**A**) and patient recommendation of the therapy (**B**) at month 6. Number of patients analyzed is 22 for intrathecal baclofen (ITB) arm and 23 for conventional medical management (CMM) arm.

More patients in the ITB arm were satisfied with the reduction in spasticity than in the CMM arm at month 6: 16/22 (73%) of ITB patients versus 11/23 (48%) of CMM patients responded agree/strongly agree (Figure 5A). Nearly twice as many patients in the ITB arm reported being highly satisfied (strongly agree) with the reduction in spasticity at month 6 compared with the CMM arm (10/22 [45%] versus 6/23 [26%]; Table II in the online-only Data Supplement). In addition, more patients in the ITB arm were willing to recommend the therapy than in the CMM arm at month 6 (16/22 [73%] versus 14/23 [61%], respectively; Figure 5B).

## Discussion

Patient-reported outcome measures are an important complement to clinical and functional outcome measures,^24^ providing a comprehensive picture of the impact of spasticity on the patient’s everyday life. Results from this analysis of patient-reported secondary outcomes from SISTERS indicate that ITB therapy over (or at) 6 months is associated with pain reduction and an overall improvement in health and well-being in PSS patients. Notably, we observed a significant effect of treatment with ITB therapy over CMM for reduction of NPRS least and actual pain. The observed pain reduction in ITB patients was apparently not because of major changes in pain medication: more patients in the ITB arm were taking pain medications at baseline, but changes in the number of patients decreasing or increasing pain medication from baseline to month 6 were comparable between the 2 treatment arms. Findings from the present study confirm and extend previous observations by Schiess et al,^11^ who reported a reduction in VAS pain measurements after 12 months of ITB treatment when compared with baseline. Our results, therefore, help address the relative paucity of evidence on pain relief with spasticity treatments, which is often not a primary treatment goal.

For NPRS worst pain, an improvement from baseline was observed in the ITB but not the CMM arm at month 3, although by month 6 the magnitude of pain reduction with ITB was less pronounced and the effect not significant between the 2 groups. Conceivably, the lack of treatment effect on worst pain could be because of less dose adjustment of ITB therapy during the latter part of the study. The study was also not powered to detect treatment effects for secondary outcomes; thus, for some measures, differences between groups may not be statistically significant despite evidence of improvement over baseline in the ITB arm.

It remains unclear whether the reduction in pain after ITB therapy in PSS patients was directly associated with the reduction in spastic hypertonia and muscle tone in the extremities^19^ as there were insufficient patient numbers to detect a correlation. It is possible that ITB may relieve spasm- or cramp-related pain as the reduction of involuntary spasms has previously been reported to decrease pain.^12^ However, spasm frequency was not determined in this study. Finally, our results are consistent with the findings of Wissel et al,^25^ who reported that poststroke patients treated for spasticity with onabotulinum toxin A showed decreases in muscle tone and pain, with no correlation found between the 2 outcomes.

Spasticity and pain can increase disability and contribute to poor QoL among stroke survivors.^6,26^ For the mean EQ-5D-3L utility score, the analysis showed a significant effect of treatment in favor of ITB by month 6, despite the relatively short follow-up period. Furthermore, although no statistically significant effect between groups was observed for the change from baseline to month 6 in the EQ-5D-VAS score, a greater numeric improvement was seen in the ITB arm during follow-up. For individual EQ-5D dimensions, the reduction in the proportion of patients reporting extreme problems on the pain/discomfort dimension at month 6 correlates with the observed improvements in NPRS pain scores. Our results agree with those from previous studies in PSS that showed an increase in QoL measures with ITB therapy. For example, Ivanhoe et al^13^ used the Sickness Impact Profile, a generic health-related QoL measure with psychosocial and physical domains (but no pain items), and noted significant improvements in mean Sickness Impact Profile scores overall and at 3 and 12 months postimplant. In addition, Schiess et al^11^ reported a statistically significant improvement between baseline and 12 months for several SS-QOL domains (family roles, mobility, personality, self-care, social roles, thinking, upper extremity function, and work/productivity). In our study, we observed a numeric improvement from baseline in the mean SS-QOL summary score for ITB patients over (or at) 6 months, with little change for CMM patients.

Consistent with the observed effects on pain reduction and QoL, a higher overall rating of patient satisfaction for spasticity reduction was reported for ITB patients versus CMM patients, despite the necessity of a surgical procedure, and patients in the ITB arm showed greater willingness to recommend the therapy. However, patients in the ITB arm had, on average, a higher number of study visits than those in the CMM arm (14 and 4 visits, respectively^19^) and the higher patient satisfaction among ITB patients may partly reflect the increased level of follow-up.

SISTERS was designed to evaluate a wide range of clinician- and patient-reported outcomes and, as such, provides the first nondescriptive evaluation of pain reduction with ITB therapy in poststroke patients. There are some limitations of this study; specifically, the small number of patients (lower than anticipated sample size owing to recruitment difficulties^19^) and the fact that it was not powered to assess secondary outcomes. Assessment of pain was limited to the NPRS; data about specific pain characteristics and pain distribution were not collected, and therefore it was not possible to further classify the type of pain. Furthermore, assessment of patient-reported outcome measures was not blinded, and all these outcomes are subjective in nature. In addition, there is a lack of data on caregiver burden. Notwithstanding these limitations, we observed a consistent trend for improvement over baseline in the ITB arm, versus little change in the CMM arm. Importantly, our study highlights the need not only to look at spasticity reduction, but also measures of pain and QoL when considering treatment options for PSS.

## Conclusions

Our results suggest that ITB delivery provides an improved therapeutic effect when compared with CMM using oral spastic medications and physiotherapy. Reduction in pain scores, improvement in QoL measures, and high patient satisfaction with therapy were demonstrated, despite the necessity of a surgical procedure. Overall, these data reinforce and expand on existing evidence in the literature and support the use of ITB therapy for treatment of generalized spasticity with associated pain and reduced QoL.

## Acknowledgments

We thank all participants and carers who participated in this study. We gratefully acknowledge the support and work of the study research team including Nathalie Berthuy and Meghann Loven (Clinical Study Managers) as well as the Investigators (see Appendix). We also acknowledge Dr Sarah Hopwood (Scinopsis, France) for medical writing assistance with this publication paid by Medtronic.

## Sources of Funding

This study was supported by Medtronic International Trading Sàrl.

## Disclosures

M. Creamer, G. Cloud, Dr Yochelson, Dr Francisco, A.B. Ward, Dr Zampolini, and Dr Saltuari report personal fees from Medtronic. J. Wissel reports personals fees from Medtronic, Allergan, Ipsen, Merz, and Sintetica. A. Abouihia and A. Calabrese are employees of Medtronic. The other author reports no conflicts.

## Supplementary Material

## Appendix

List of Investigators: Drs R. Van der Looven and K. Bouche (UZ Gent, Gent, Belgium), Dr B. Rubin (Design Neuroscience Center, Doral, FL), Dr S. Khurana (University of Miami, Miami, FL), Dr S. Moraleda (Hospital Universitario La Paz, Madrid, Spain), Dr A. Bender (Therapiezentrum Burgau, Burgau, Germany), Dr J. Ruijgrok (AZ Maastricht, Maastricht, the Netherlands and Hôpital fribourgeois Meyriez-Murten, Switzerland), Dr J. Shilt (Saint Alphonsus Regional Medical Center, Boise, ID and Texas Children’s Hospital - The Woodlands, TX), T. Lejeune (UCL Saint-Luc, Brussels, Belgium), Dr M. Ebke (Rhein-Sieg-Klinik Dr Becker Klinikgesellschaft, Nümbrecht, Germany), Dr F. Molteni (Villa Beretta, Costamasnaga, Lecco, Italy), Dr M. Lawson (Tallahassee Neurological Clinic, Tallahassee, FL), Dr K. Grabljevec (Univerzitetni Rehabilitacijski Institut Soca, Ljubljana, Slovenia), Dr C. Dalton (St George’s Hospital, Tooting, London, United Kingdom), and Dr H. Matzak (Landeskrankenhaus Hochzirl, Zirl, Austria).
