# Supplementary material for: Effect of Intrathecal Baclofen on Pain and Quality of Life in Poststroke Spasticity: A Randomized Trial (SISTERS)
Source: Stroke. 2018 Aug 14;49(9):2129–37. doi: 10.1161/STROKEAHA.118.022255 (PMC6116794; doi:10.1161/STROKEAHA.118.022255)
Supplement: Supplementary file 1 [file str-49-2129-s001.pdf]

SUPPLEMENTAL MATERIAL

Supplement to: Creamer, M et al: Effect of intrathecal baclofen on pain and quality of life in post-stroke spasticity: a randomized trial (SISTERS)

Supplemental Tables

Table I. Inclusion/exclusion criteria for SISTERS

**Inclusion criteria**
1. Patient (or legal guardian) has been informed of the study procedures and has given written informed consent.
2. 18-75 years of age.
3. Patient experienced last stroke >6 months prior to enrollment.
4. Patient presents spasticity in at least 2 extremities.
5. Patient presents an Ashworth score ≥3 in a minimum of two of the affected muscle groups in the lower extremity.
6. Patient is eligible to receive ITB therapy following the Adult Spasticity Algorithm:<br>a. patient does not reach his/her therapy goal with other treatment interventions.
7. Stable blood pressure:<br>a. no change in hypertensive medication in last month<br>NOTE: ventriculo-peritoneal shunts and valves could be present.
8. If female, she must either:<br>a. be post-menopausal or surgically sterilized; or<br>b. use a hormonal contraceptive, intra-uterine device, diaphragm with spermicide, or condom with spermicide, for the duration of the study.
9. Patient/family is willing to comply with study protocol including attending the study visits.
**Exclusion criteria**
1. Patient/family is considered by the physician to be unable or unwilling to participate in long-term ITB therapy management.
2. Patient has known hypersensitivity to baclofen.
3. Active systemic infection.<br>NOTE: pressure sores were not a contraindication unless they were present near the implant sites.
4. Presence of a cardiac pacemaker, implantable cardioverter defibrillator (ICD), implantable neurostimulator, or drug delivery device.
5. Uncontrolled refractory epilepsy.
6. Use of oral vitamin K antagonists, e.g. warfarin/coumadin; unless the patient can switch to another accepted anticoagulant (e.g. heparin, aggrenox, fragmin, plavix, ticlid) for the period of the ITB test and implant.
7. Patient is pregnant or breast-feeding[a].
8. Patient received a botulinum toxin injection less than 4 months ago.
[a] Confirmation that the patient was not pregnant had to be established by a negative urine pregnancy test at baseline. A pregnancy test was not required if the patient was postmenopausal or surgically sterilized.

Table II. Likert scale responses for patient satisfaction with the therapy at Month 6 (ITT population)

Patient satisfaction with spasticity reduction[a]	ITB (N=31)	CMM (N=29)
N	22	23
Strongly disagree (1)	1 (4.5)	4 (17.4)
Disagree (2)	1 (4.5)	3 (13.0)
Neither agree nor disagree (3)	4 (18.2)	5 (21.7)
Agree (4)	6 (27.3)	5 (21.7)
Strongly agree (5)	10 (45.5)	6 (26.1)
**Patient therapy recommendation[b]**	**ITB (N=31)**	**CMM (N=29)**
N	22	23
Strongly disagree (1)	2 (9.1)	2 (8.7)
Disagree (2)	1 (4.5)	3 (13.0)
Neither agree nor disagree (3)	3 (13.6)	4 (17.4)
Agree (4)	7 (31.8)	5 (21.7)
Strongly agree (5)	9 (40.9)	9 (39.1)

All values are number (%) of patients unless indicated otherwise.

a: Patients were presented with the following statement in their native language to assess their satisfaction with their respective treatment (ITB therapy or CMM): “I am satisfied with the reduction in spasticity provided by my treatment.”

b: Patients were presented with the following statement in their native language to assess their satisfaction with their respective treatment (ITB therapy or CMM): “I would recommend this therapy to a friend.”

Responses were assessed using the 5-level Likert Scale, which measures either positive or negative response to a statement, with 1 representing “strongly disagree” and 5 representing “strongly agree”.

CMM indicates conventional medical management; ITB, intrathecal baclofen; ITT, intent-to-treat.
